# News and Coming Events

**Published:** 1908-05-09

**Authors:** 


### NEWS AND COMING EVENTS.

The next session of the General Medical Council will begin on Tuesday, May 26.

His Grace the Duke of Northumberland will preside at the annual dinner of the Royal Sanitary Institute on May 12 at the Langham Hotel.

The anniversary dinner of King's College Hospital will take place at 7 for 7.30 p.m., in the Grand Hall, Hôtel Cecil, on May 11, under the chairmanship of his Excellency the American Ambassador (the Hon. Whitelaw Reid).

Professor M. McHardy presided at the annual meeting of the Royal Eye Hospital, which this year celebrates its jubilee. The maximum accommodation for which the hospital was built was 20,000. Owing to the great increase in the number of patients the Council are considering further extensions.

Dr. J. T. Tasker-Evans, upon his retirement after twenty-nine years' service as honorary medical officer, has been presented with a silver tea and coffee service and a cheque for forty guineas from the governors and supporters of the Hertford General Infirmary.

Sir James Crichton-Browne, M.D., has been re-elected Treasurer of the Royal Institution for the ensuing year. Sir Thomas Barlow, M.D., and Dr. Donald Hood have been elected Managers; and Mr. C. A. Ballance and Dr. J. Dundas Grant, Visitors of the Institution.

The Murchison Scholarship, founded in 1880, to perpetuate the memory of Dr. Charles Murchison, awarded alternately by the University of Edinburgh and the Royal College of Physicians of London, has been awarded by the College to E. L. M. Lobb, of Guy's Hospital.

The festival dinner in aid of the Rebuilding Fund of the Hospital for Women, Soho Square, will be held on Monday, May 11, at the Whitehall Rooms, Hôtel Métropole, at 7 for 7.30 p.m. The Earl of Shaftesbury, President of the Hospital, will be in the chair.

The annual meeting of the Governors of the West London Hospital was held on April 30, the Duke of Abercorn, the chairman of the Board of Management, presiding. The Duke of Abercorn was unanimously elected President of the hospital in succession to the late Duke of Devonshire. He will still retain the position of chairman of the Board of Management.

The death is announced from Paris of Professor Chamberland, the physiologist, who was assistant director of the Pasteur Institute and a member of the Academy of Medicine. He took part in the researches of M. Pasteur into the nature of fermentation and the causes of anthrax and rabies, and was the author of several works dealing with these subjects.

The arrangements for the International First Aid Congress at Frankfürt-on-Maine, to be held during Whitsun week, June 9 to 13, are progressing. It is believed that the attendance will exceed 700. The hon. committee includes the German Imperial Chancellor (Prince Bülow) and representatives of the departments of the German Union of States. At a committee meeting of the British section, presided over by the Duke of Marlborough (who has stated his intention of being present at Frankfürt), it was stated that Great Britain would be well represented. The Hon. Secretary is Mr. S. Osborn, F.R.C.S. of Datchet, near Windsor.

Mr. Stephen Paget, Honorary Secretary of the Research Defence Society, in a letter to the *Times* of May 6, states that since the publication of Lord Cromer's letter more than 200 members have joined the Society, which now numbers 1,026 members, of whom 73 are ladies. Branch societies are already being formed in many towns.

Dr. E. H. Lloyd, M.B., F.R.C.S., retired Army Brigade Surgeon, died suddenly, at the age of 72, on May 3. Dr. Lloyd joined the Army as an assistant-surgeon in 1859, was promoted to the rank of surgeon in 1873, and to that of surgeon-major in 1875, in which year he retired on half pay. He served in the Nile Expedition in 1884-5, and was in charge of the field hospital at Kaibar.

The annual meeting of the governors of the Metropolitan Hospital was held on May 4, under the presidency of Mr. C. J. Thomas. The report stated that the work of the hospital during the past year had steadily increased in every direction. There was a deficiency of £1,940 in the ordinary income over that of the preceding year, while the total expenditure amounted to £14,653, as compared with £14,313 in 1906.

Mr. Henry Weedon, Lord Mayor of Melbourne, on May 1 opened at the Agricultural Hall, Islington, a Municipal Building and Public Health Exhibition. Captain H. Riall Sankey, chairman of the Honorary Advisory Council of the Exhibition, gave particulars as to the many municipalities contributing exhibits. These included the Municipality of Lyons (France), which sent plans of the public slaughter-houses and of the schools. The Exhibition will remain open until May 12.

Her Majesty the Queen has consented to open, on some day in June to be fixed later, the new out-patient department of the Hospital for Sick Children, Great Ormond Street, which has recently been built by Mr. W. Astor as a memorial to his late daughter. The Hospital for Sick Children was founded on a small scale by Dr. Charles West and a few others 56 years ago, and was the first hospital established in this country for children. It is also now the largest children's hospital in the British Empire.

Professor William Osler, M.D., F.R.S., delivered the Linacre Lecture on Wednesday, May 6, in the New Museum of the University of Cambridge. The subject was: "Thomas Linacre: his Life and Works." The Linacre Lectureship was founded at St. John's College in 1552. It has hitherto been tenable by one occupant for a number of years; but, since the retirement of the last lecturer, Dr. Donald Macalister, on his appointment to the Principalship of the University of Glasgow, it has been converted into an annual appointment, to be held each year by some distinguished member of the medical profession.

The Medico-Legal Society will hold a meeting on Friday, June 5, in Edinburgh. A discussion will be opened by Mr. R. W. Renton on English and Scottish procedure in the preliminary investigation of sudden or violent deaths. Dr. Urquhart will read a paper on certain imperfections in the law in connection with insanity, and demonstrations will be given by Professor Harvey Littlejohn, Dr. Harvey Pirie, Sir Henry Littlejohn, and others. On Saturday the members will, at the invitation of Professor Glaister, visit the new forensic medicine and public health department of the University of Glasgow. Further particulars may be obtained from Dr. Stanley B. Atkinson, 10 Adelphi Terrace, London, W.C.

The Bishop of Bath and Wells preached the annual Spital sermon to the governors of the Royal Hospitals at Christ Church, Newgate Street, on the afternoon of April 29. The hospitals are Christ's Hospital, Bridewell Hospital, St. Bartholomew's, and St. Thomas's, and in virtue of their office as governors the Lord Mayor, the City Sheriffs, and members of the Corporation attended in state.

The Duchess of Montrose performed the opening ceremony on April 29 at the new Glasgow Maternity and Women's Hospital, which has been built and equipped at a cost of £80,000. Principal Macalister said that the institution was a splendid addition to the equipment of the Glasgow Medical School, and Sir J. Halliday Croom pronounced it second to none of the hospitals he had seen in this country. The Lord Provost appealed strongly for increased financial support, the sum of £30,000 being still required to free the institution from debt.

The governors of the Miller Hospital and Royal Kent Dispensary, who have re-elected Lord Dartmouth as President, have decided to alter the name of the institution to the Miller General Hospital for South-East London (with which is incorporated the Royal Kent Dispensary). It is hoped that during the present year some practical steps may be taken for the extension of the hospital, which serves a very large district in South-East London, and in which last year 17,432 patients received attention.

The Council of the University of Manchester having decided on the appointment of a second professor in the department of medicine, one of the chairs will be devoted to clinical medicine and the other to systematic medicine. Dr. Graham Steell, now Professor of Medicine in the University and senior physician in the Royal Infirmary, will hold the chair of clinical medicine, and Dr. George R. Murray, who until recently held the Heath Professorship of Comparative Pathology in the Durham University, has been appointed professor of systematic medicine. Under the agreement entered into between the University and the Royal Infirmary, Dr. Murray becomes on his appointment a member of the honorary staff of the Royal Infirmary. It is expected that he will undertake the duties of the chair in the University at the beginning of next session, and at the Infirmary when the new buildings are opened.

A festival dinner in aid of the funds of the City of London Hospital for Diseases of the Chest (Victoria Park Hospital) will take place at the Trocadero Restaurant, Piccadilly Circus, on Tuesday, May 12, at 7 for 7.30 p.m., Mr. Herbert Robertson, Chairman of the House Committee) in the chair.

The annual general meeting of the Medical Defence Union will be held on May 21 at the Medical Institution, Hope Street, Mount Pleasant, Liverpool, at 4.30 p.m. The annual report of the Council, together with the balance sheet and accounts as certified by the auditors, will be presented to the members, and the usual statutory business carried out.

Brigade Surgeon William Pierce Kelly, F.R.C.S., late of the Indian Medical Service, and Inspector-General of Prisons in Burma, died on May 2 in Dublin. He served in the Indian Mutiny Campaign in 1858, and with the North Canara Field Forces, receiving the Mutiny medal.

The new wing of the Evelina Hospital for Sick Children, Southwark Bridge Road, is now complete and in full use. It contains the medical department and dispensary on the ground floor; on the first floor, surgical cases are examined; on the second and third floors accommodation has been provided for the matron, sisters, and nurses. An outside escape staircase has also been built. The total cost of the additions has been £14,000, towards which King Edward's Hospital Fund contributed £2,250, and £5,000 was received as a Lewis legacy. Only £3,000 now remains to be found.

The Court of Appeal, composed of Sir Gorell Barnes and Lord Justices Moulton and Farwell, gave judgment on May 4 on the appeal of the Malvern Urban District Council from the judgment in the action brought against them by Dr. John Campbell Fergusson, proprietor of a hydropathic establishment at Malvern. At the recent trial of the action before Mr. Justice Lawrance and a special jury judgment was entered for the plaintiff for £7,500 and costs. Dr. Fergusson sued the local authority for damages for having, as he alleged, permitted sewage to contaminate water which he used for the institution, and which caused, he said, a number of cases of typhoid fever, in respect of which he had been called upon to pay large sums as damages. The appeal was allowed, with costs.

